# Microneedle patch tattoos

**DOI:** 10.1016/j.isci.2022.105014

**Published:** 2022-09-14

**Authors:** Song Li, Youngeun Kim, Jeong Woo Lee, Mark R. Prausnitz

**Affiliations:** 1 School of Chemical and Biomolecular Engineering, Georgia Institute of Technology, Atlanta, GA 30332, USA

**Keywords:** Medical device, Medical biotechnology, Biomaterials

## Abstract

Medical tattoos provide medical information, guide radiotherapy, and improve cosmetic outcomes of medical interventions. These tattoos are administered by repeated needle injection that causes pain, bleeding, and risk of infection, which limit more widespread use. Here, we developed single-use microneedle (MN) patches to deposit tattoos in the skin in a simple, rapid, painless, and bloodless way without biohazardous sharps waste. MN patch tattoos were designed with numbers, letters, symbols, environmentally responsive inks, and QR codes. Colored tattoos, and tattoos only visible with ultraviolet illumination for increased privacy, were developed and retained in the skin for at least one year. These MN patch tattoos recorded medical conditions such as diabetic medical alerts and vaccination status, responded to biophysical cues for possible physiological monitoring, and encoded complex personal health information. MN patches may increase safety and access to medical tattoos for improved fiducial marking, medical information storage, physiological monitoring, and cosmetic outcomes.

## Highlights

• Microneedle patches tattoo skin in a simple, rapid, painless, and bloodless way
• Microneedle patches facilitate medical, veterinary, and cosmetic tattooing
• Medical tattoos communicate health status and store information in a patient’s skin

## Graphical abstract

**Figure:**
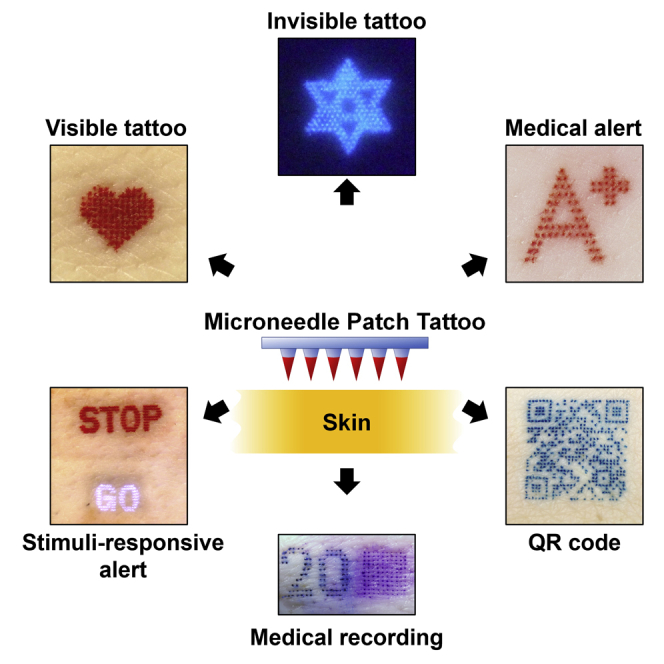

## Introduction

Tattoos have been used in human societies and cultures around the world for millennia as a form of body modification that provides art, information, spiritual meaning, or other attributes (Krutak, 2015). From very early human history, tattoos have not only been associated with cultural or religious signs (Kosut, 2000) but also have had intended medical uses (Krutak, 2013). The oldest known tattoos are believed to be those found on mummies from up to 5,000 years ago (Deter-Wolf et al., 2016). These tattoos are believed to have had therapeutic purpose, such as marking acupuncture sites or alleviating pain or other ailments (Krutak, 2013).

Nowadays, tattoos are in widespread use, both for medical or veterinary applications as well as for ornamental or cosmetic purposes (Kluger and De Cuyper, 2018; Vassileva and Hristakieva, 2007). For example, medical tattoos are used to communicate chronic or life-threatening medical conditions such as diabetes to emergency personnel (Kluger and Aldasouqi, 2013), to identify body locations for repeated therapeutic treatment such as radiotherapy (Landeg et al., 2016) or to treat cosmetic outcomes of medical conditions such as dermatographic color correction of vitiligo (Singh and Karki, 2010) and simulating areola in nipple-areola reconstruction therapy (Vandeweyer, 2003). Animals are tattooed to indicate sterilization status (Griffin et al., 2016, 2020) or for individual identification and/or population monitoring of free-roaming animals (ICAM, 2017). Outside of medicine, tattoos are also in widespread use for cosmetic decoration or self-expression, with 10%–20% of the population in countries around the world having tattoos (Heywood et al., 2012; Kluger, 2015; Laumann and Derick, 2006). Incidence is increasing over time and is higher among those under age 40 years.

Tattoos are typically made by injecting pigments or inks into the skin using needles or lancets. Modern tattoo machines use electromagnetic force to move the needles in and out of the skin repeatedly at high frequency up to several thousand times per minute to deposit material in the skin (Oyarte Gálvez et al., 2019; Rosenkilde, 2015), and the needles can penetrate the skin to a depth from several hundred micrometers up to around 2 mm (Barua, 2015; Sardana et al., 2015). The liquid tattoo ink is guided via the needle and deposited in the skin. Although many different tattooing methods have been developed throughout human history, the basic principle of introducing colored particles into the skin by needle puncture is largely unchanged.

Adverse effects of tattooing have been reported, with incidence as high as 31% (Antoszewski et al., 2006) and 68% (Klügl et al., 2010) describing skin complications after receiving a tattoo, most commonly pruritus and/or bleeding (Paola et al., 2016). There is also a risk of infection, which is primarily bacterial and localized to the skin, with an incidence of up to 5% of tattoo recipients (Klügl et al., 2010; Laux et al., 2016). Infection is believed to be due largely to contaminated ink or needles due to poor manufacturing or unsafe practices during tattoo application (e.g., use of saliva as a needle lubricant or tap water as an ink diluent) (Laux et al., 2016; Paola et al., 2016).

Tattooing has technical similarities to hypodermic injection in medicine, and tattoo tools have been adapted for drug delivery applications (Pokorna et al., 2008; Shio et al., 2014). Likewise, advances in drug delivery may be used to improve tattoo administration. In the biomedical field, microneedle (MN) patches have been developed as an alternative to injections that enable simplified administration for targeted delivery to the skin using a skin patch (Ingrole et al., 2021; Prausnitz, 2017). When these patches are pressed to the skin, microscopic needles penetrate below the skin surface to release coated or encapsulated drug into the skin for local effects or systemic uptake via dermal vasculature.

MN patches have been widely studied for minimally invasive drug delivery that causes little or no pain or bleeding, generates no biohazardous sharps waste, and can be administered after only brief training (Duarah et al., 2019; Ingrole et al., 2021; Ita, 2017; Norman et al., 2014; Prausnitz, 2017). MN patches can be manufactured at low cost as single-use, sterile devices. The MNs on these patches are generally hundreds of micrometers long, which can effectively penetrate the skin to release payloads in the dermis, suggesting that MN patches may be able to simplify tattoo administration to increase access, increase safety, and decrease cost.

In this study, we designed MN patches and fabrication techniques to produce skin tattoos, in which each MN represents a dot or pixel of the tattoo image. Using single-color, multi-color, changing-color, and ultraviolet (UV)-visible tattoo inks, we made MN patches with tattoo symbols, numbers, letters, and other medical/decorative images applied to the skin.

## Results and discussion

### Symbol tattoos by MN patches

Conventional MN patches for drug delivery are typically arranged as a square or circular array of MNs. To create MN patches with other shapes that can communicate visual information, we used a CO_2_ laser cutter to drill conical cavities in polydimethylsiloxane (PDMS) sheets to form molds with any desired pattern. In this way, we made MN patches in which each MN behaved like a pixel or dot that together created the tattoo shape or image. Each MN was made of a mixture of tattoo ink particles and a biocompatible, water-soluble polymer (i.e., poly(acrylic acid), PAA).

**Figure 1:**
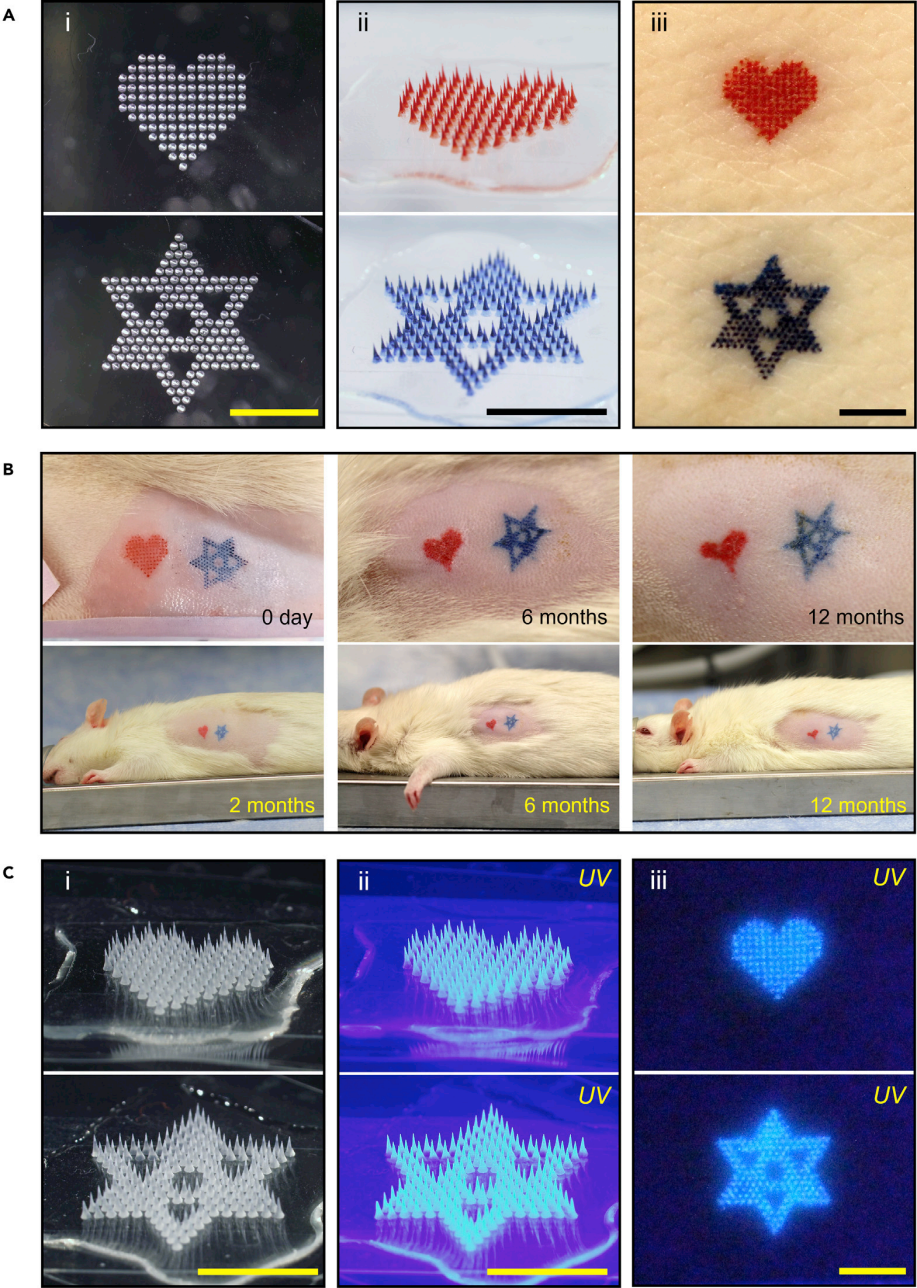
Symbol tattoos by MN patches (A) Representative microscopy images of red-heart and blue-star MN patch tattoos: (i) top view of the PDMS molds with the tattoo patterns; (ii) MN patches loaded with red or blue tattoo ink; (iii) porcine skin tattooed by the MN patches *ex vivo*. Scale bars: 5 mm. (B) Representative photographic images of red-heart and blue-star tattoos administered to rats by MN patches imaged over time for up to one year. (C) Representative microscopy images of UV-visible heart and star MN patch tattoos: (i) UV-MN patches loaded with UV-visible tattoo ink, seen in bright field; (ii) UV-MN patches illuminated by UV light, showing blue fluorescence of the UV tattoo ink; (iii) porcine skin tattooed by the UV-MN patches *ex vivo*, seen with illumination by a UV flashlight. Scale bars: 5 mm.

To demonstrate this approach, we prepared molds in the shape of a heart and a hexagon star symbol (Figure 1A(i)) that were used to make MN patches containing tattoo ink in the same pattern (Figure 1A(ii)). When pressed to the skin, the tattoo ink was deposited by the MNs in the skin to transfer the heart and star patterns as skin tattoos (Figure 1A(iii)). Ink deposition in the skin was accompanied by dissolution of the water-soluble MN, which left behind a used patch backing with no biohazardous sharps (Figure S1 in supplemental information).

The red-heart and blue-star MN patches were applied to rats to assess MN patch tattooing *in vivo* (Figure 1B). These tattoo images were transferred to the skin and remained visible for at least one year, maintaining both their shape and color. The procedure was well tolerated, with no adverse reactions seen in the skin. The red-heart image exhibited some distortion after one year, which may have been caused by body shape change or skin aging, which is known to affect tattoos in humans (Hudson, 2009). In addition, the quick metabolism and growth of rats may play a role, because one year is a large fraction of a rat’s total lifespan (Sengupta, 2013). Overall, these findings demonstrate the feasibility of tattooing by MN patches.

Similarly, heart and star tattoo MN patches were made by loading UV fluorescent tattoo ink that could not be seen in ambient light (Figure 1C(i)) but fluorescent blue when illuminated with UV light (Figure 1C(ii)). Upon application to the skin, the UV tattoos were visible with UV illumination by a handheld UV flashlight, thereby helping to keep the tattoo private (Figure 1C(iii)).

### Medical tattoos by MN patches

**Figure 2:**
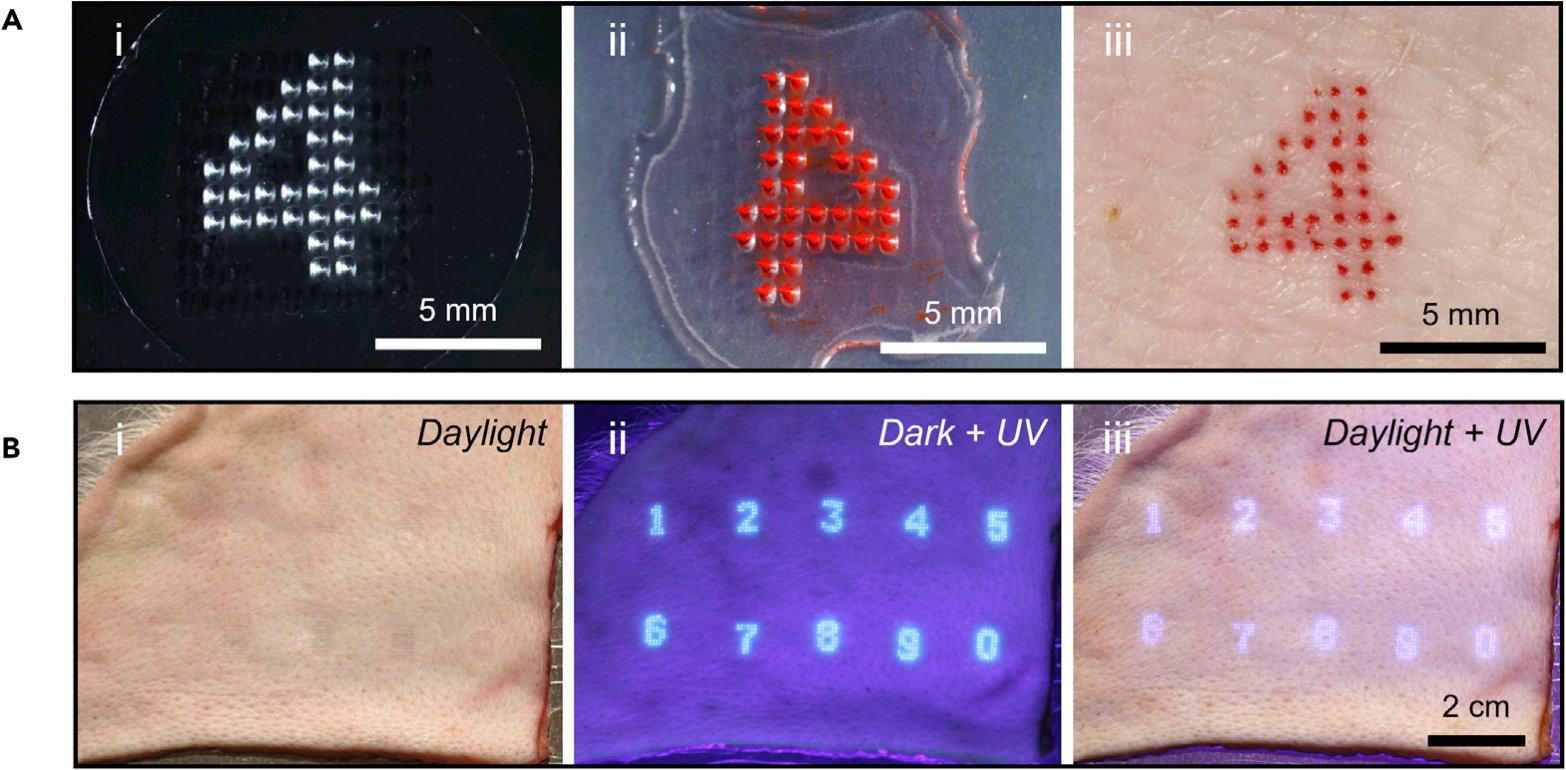
Number tattoos by MN patches (A) Representative microscopy images of a MN patch tattoo with the number “4”: (i) top view of the PDMS mold with a “4” pattern; (ii) MN patch mold loaded with red tattoo ink; (iii) porcine skin tattooed by the MN patch *ex vivo*. (B) Representative photographic images of porcine skin tattooed with numbers from “0” to “9” by UV-MN patches, showing that the tattoos were invisible in daylight (i). The tattoos became visible when illuminated with UV light in darkness (ii) and in daylight (iii).

We next sought to develop the MN technology in a way that could benefit medical and veterinary tattoos to contain information that can guide treatment. In some cases, this would require using letters and/or numbers to record medical information. As an example, we made MN patches to tattoo the number “4” and applied it to skin (Figure 2A). Note that the mold (Figure 2A(i)) and skin tattoo (Figure 2A(iii)) show the number “4” in the correct orientation, but the MN patch when viewed with the MNs pointing up shows the mirror image (Figure 2A(ii)).

Building off the single-digit tattoo, we next made MN patch tattoos for all 10 digits from 0 to 9 in the skin, this time using UV-fluorescent ink (Figure 2B). These UV tattoos were invisible in the skin in daylight (Figure 2B(i)) but became visible when illuminated with UV light either in darkness (Figure 2B(ii)) or in daylight (Figure 2B(iii)). Such numerical tattoos could be used to record the date or year of a medical intervention or other quantitative information needed for treatment or monitoring.

**Figure 3:**
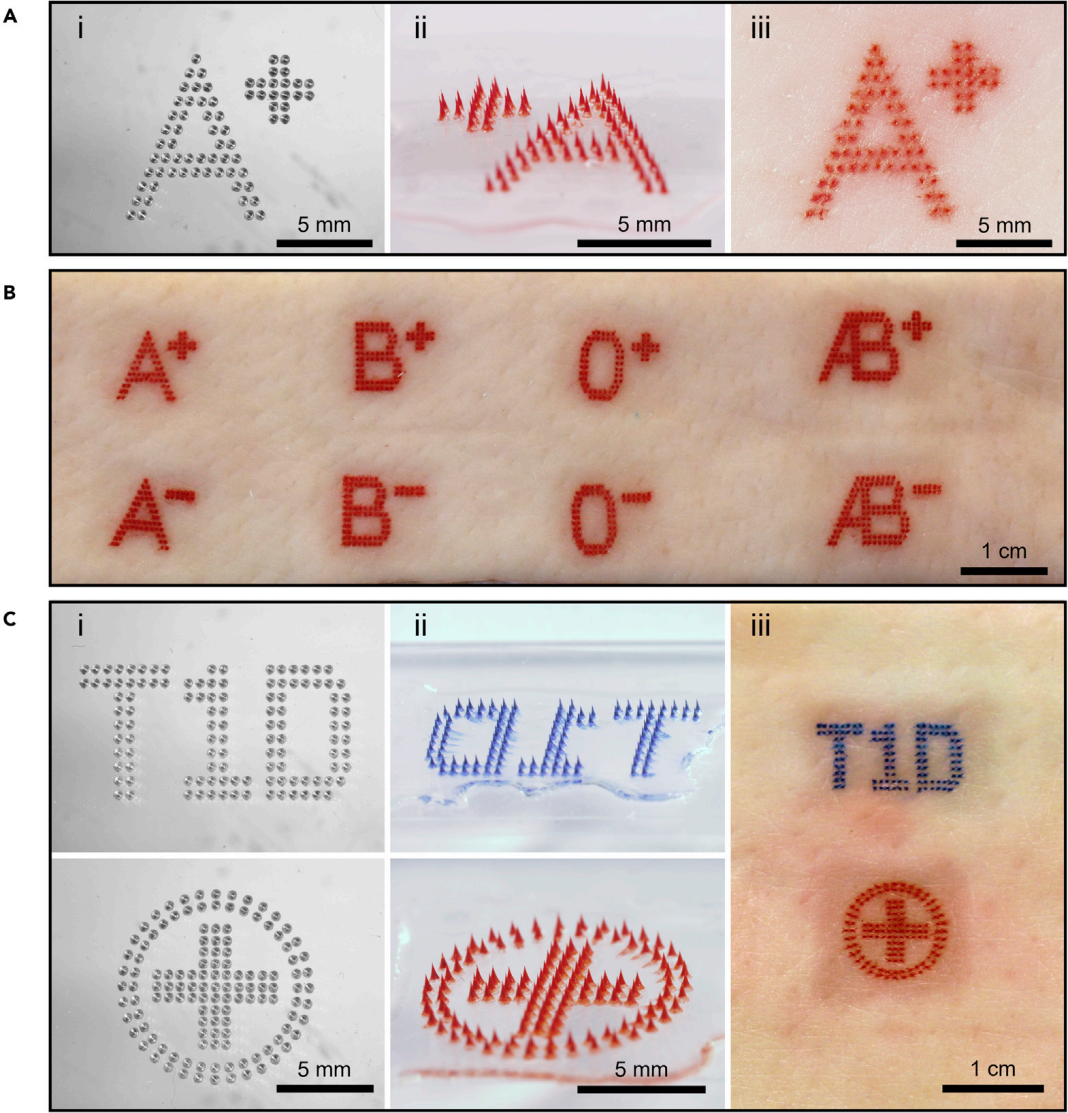
Medical tattoos by MN patches (A) Representative microscopy images of a MN patch tattoo with an “A+” label indicating blood type: (i) top view of the PDMS mold with an “A+” pattern; (ii) MN patch loaded with red tattoo ink; (iii) porcine skin tattooed by the MN patch *ex vivo*. (B) Representative photographic images of porcine skin *ex vivo* tattooed with eight different blood-type labels by MN patches. (C) Representative microscopy images of MN patch tattoos with “T1D” (type-1 diabetes) and red-cross medical alert symbols: (i) top view of the PDMS molds with medical alert patterns; (ii) MN patches loaded with blue or red tattoo ink; (iii) porcine skin tattooed with type-1 diabetes medical alert symbols by MN patches *ex vivo*.

Medical tattoos might also benefit from other symbols besides numbers. As an example, we made MN patches to tattoo skin with the eight different blood-type codes (Figures 3A and 3B). As another example, a diabetes alert tattoo consisting of a blue “T1D” label (i.e., type-1 diabetes) and a red-cross symbol was also enabled by MN patches (Figure 3C). We incorporated two different colors in this tattoo by filling different tattoo inks into the MN patches.

These medical tattoos demonstrate the feasibility of developing MN patches to provide information using different numbers, letters, symbols, colors, and their combinations. Because these medical tattoo patches can be easily administered and read, they can enable patients to provide valuable medical information to health care personnel, such as first responders in an emergency involving, for example, extensive blood loss or a severe hypoglycemic event where the patient may not be responsive or fully coherent, as is done today, for example, using medical alert bracelets (Kluger and Aldasouqi, 2013).

### Two-component MN patches for vaccination and medical recording

**Figure 4:**
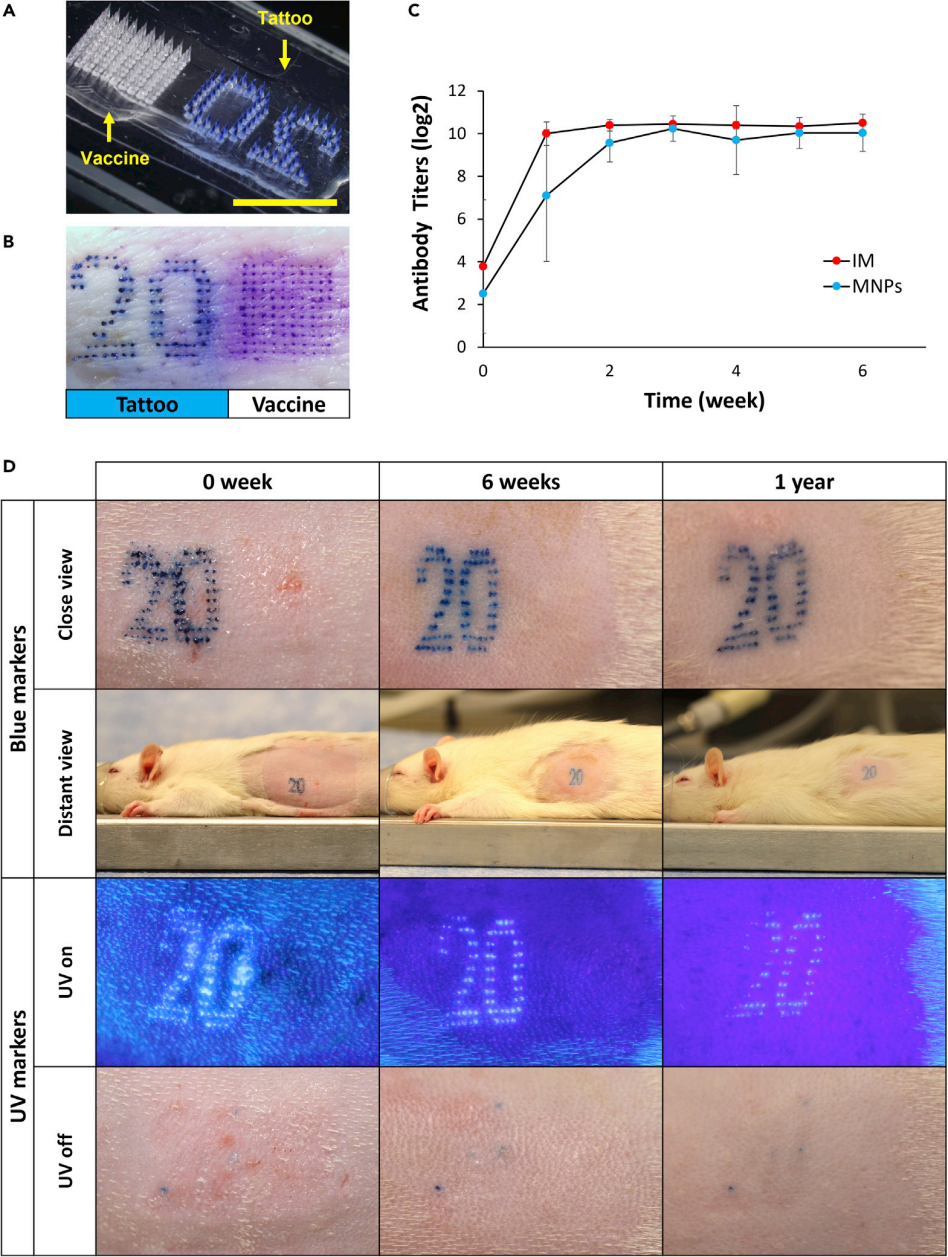
Two-component MN patches for vaccination and medical recording (A) Representative photographic image of a two-component MN patch loaded with IPV vaccine (left) and blue tattoo ink (right). Scale bar: 1 cm. (B) Representative microscopic image of porcine skin after applying a two-component MN patch *ex vivo*, showing the blue number “20” tattoo and an adjacent array of micropores stained by Gentian violet to facilitate imaging of puncture sites by the MNs containing vaccine. (C) Neutralizing antibody titers measured after IPV vaccination by two-component MN patch or IM injection of the same dose (8 DU) of the same vaccine (IPV type 2) in Wistar rats (Data was shown as mean ± S.D. N = 5–6 independent replicates per group). (D) Representative photographic images of blue and UV number “20” tattoos on rats administered by two-component MN patches were recognizable after one year to record the vaccination year 2020. Imaging was performed close (∼20 cm from skin) and distant (∼1 m from skin) with ambient lighting or with UV illumination.

The conventional use of MN patches to administer drugs and vaccines presents a dual-use scenario, where a MN patch can be used for both drug delivery and medical recording (McHugh et al., 2019). To demonstrate this concept, we developed a MN patch to administer inactivated polio vaccine (IPV) and tattoo ink in the shape of the number “20” (Figure 4A). In this way, a single MN patch could be used to vaccinate as well as leave a small marking on the skin indicating the vaccination year (i.e., 2020). This could be especially valuable in low-resource settings with limited infrastructure for health care records.

In our approach, the vaccine and tattoo ink were loaded into separate sections of the MN patch, which avoided possible negative interactions between the vaccine and ink. This simplified the MN patch design and fabrication process, but in other case the vaccine and tattoo ink could be combined in the same MNs by developing a MN patch formulation to stabilize both the vaccine and tattoo ink. MNs in this two-component MN patch effectively penetrated the skin, as indicated by the blue “20” tattoo on one side of the skin and an array of purple dots formed on the other side of the skin by a dye that selectively stains sites of skin penetration, indicating successful puncture by the IPV-containing MNs (i.e., this dye was added only for imaging purposes and would not be used during a conventional vaccination [Figure 4B]).

Immunogenicity of the IPV vaccination was assessed in Wistar rats by vaccination using either two-component MN patches or intramuscular (IM) injection of the same dose of IPV type 2. The immune response following vaccination revealed that the neutralizing antibody response to vaccination was not significantly different in the MN patch and IM vaccination groups over the course of the six-week study (two-tailed Student’s t test, p > 0.05, Figure 4C).

We also assessed the companion tattoo administered to the rats with either light-visible or UV-visible ink. The tattooed number “20” remained visible in the skin for at least one year after vaccination, i.e., the blue ink tattoo was always visible and the UV ink tattoo remained visible with UV light illumination but was invisible just in daylight (Figure 4D). No adverse effects were seen in the skin, and the procedure was overall well tolerated by the rats. The UV ink allowed the tattoo to be discreet, only to be seen with UV illumination. Still greater privacy could be enabled by using dye that is only visible in the infrared or other parts of the electromagnetic spectrum and would require specialized equipment to detect (McHugh et al., 2019).

### Responsive tattoos by light-sensitive MN patches and thermosensitive MN patches

**Figure 5:**
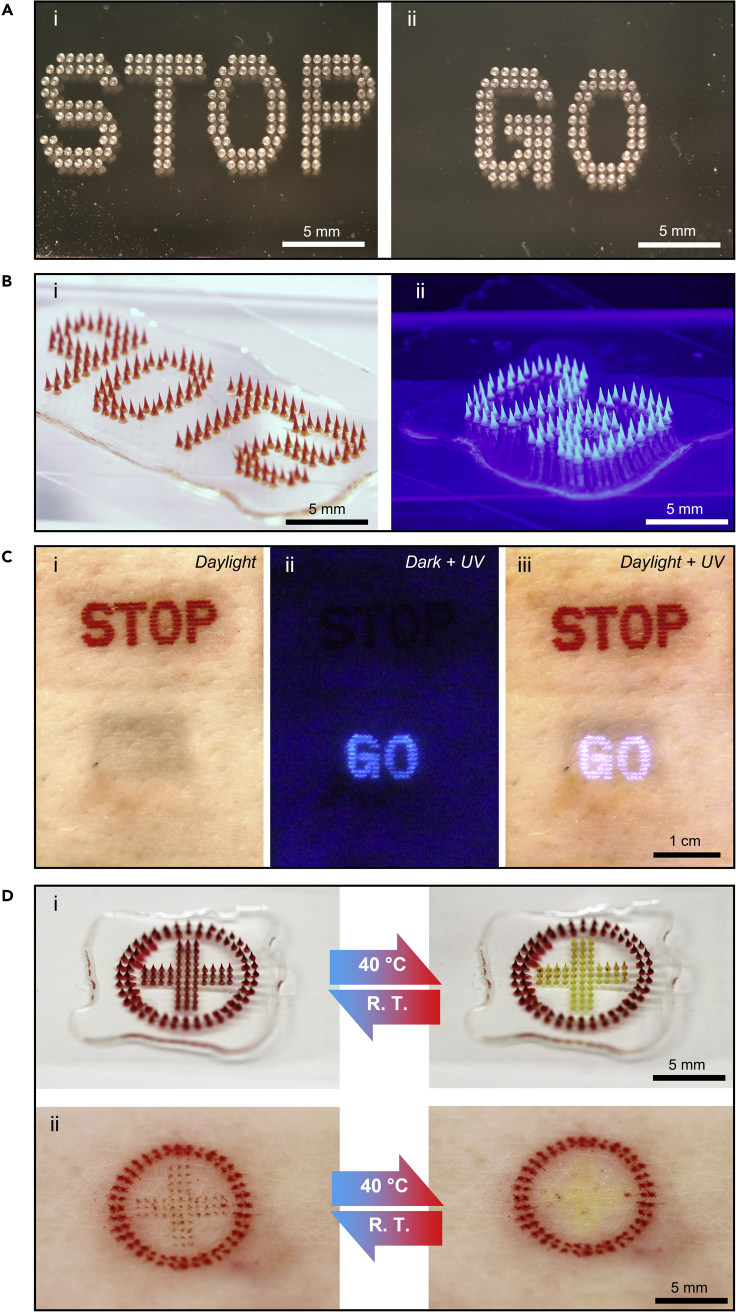
Responsive tattoos by light-sensitive and thermosensitive MN patches Representative microscopic and photographic images showing (A) a top view of PDMS molds with (i) “STOP” and (ii) “GO” patterns; (B) MN patches with a (i) “STOP” pattern loaded with red tattoo ink imaged in bright field and (ii) “GO” pattern loaded with UV-visible tattoo ink seen with UV illumination; and (C) porcine skin with responsive tattoos *ex vivo* showing (i) a red “STOP” label visible under daylight, (ii) a fluorescent “GO” label visible under UV illumination, and (iii) both “STOP” and “GO” labels visible under daylight with UV illumination. (D) Representative microscopic images of (i) a MN patch loaded with thermochromic pigments showing a red cross at room temperature that reversibly turned yellow upon heating to 40 °C, while the outer circle loaded with non-responsive red ink did not change color, and (ii) porcine skin tattooed *ex vivo* showing the red cross label reversibly turned from red at room temperature to yellow when heated to 40°C.

In some cases, it may be useful to have tattoos that are responsive to environmental changes and thereby provide different information in different settings. As an example, we combined light-visible and UV-visible tattoo inks in MN patches to create a tattoo with differential information based on lighting conditions (Figure 5). In daylight, a red “STOP” label was evident on the skin (Figure 5C(i)), whereas a blue “GO” label was seen in darkness with UV illumination (Figure 5C(ii)). Both labels were evident during UV illumination in daylight (Figure 5C(iii)).

In another example, two different inks were used to form a red-cross pattern (Figure 5D). The inner cross was made using thermochromic ink that changed color in response to a temperature change, and the outer circle was made using conventional red tattoo ink. In this way, the inner cross could change color between red and yellow reversibly upon heating across a threshold of 40°C both in the MN patch (Figure 5D(i)) and in the skin tattoo (Figure 5D(ii)). In the skin, the yellow color was almost invisible because of the skin tone. This type of tattoo may be used to monitor the temperature in the skin, such as during fever or during a treatment involving exposure to elevated temperature. Other thermochromic inks can change to different colors (Figure S2 in supplemental information) or at different temperatures, which could enable more color-change options for thermosensitive-MN patch tattoos.

Although light and thermal stimuli serve as examples in this study, prior research has demonstrated novel chemistries and technologies for materials that respond to a broad range of different stimuli of interest in medicine and other applications, including glucose, enzymes, and pH (Deirram et al., 2019; Kowalski et al., 2018; Linsley and Wu, 2017; Mu et al., 2018; Municoy et al., 2020; Ward and Georgiou, 2011; Wu et al., 2011). These functional materials and particles may be loaded into MN patches and delivered into the skin to monitor a variety of different physiological parameters (Babity et al., 2022; He et al., 2021).

### QR code tattoos by MN patch

We further increased the complexity of the tattoo pattern on MN patches by including more MNs per patch and designing the position of each MN to assemble a QR code pattern. A typical QR code consists of dark-color squares (called modules) on a white background. Its grid pattern stores digitized data, which can be text (e.g., with medical information) or internet links (e.g., to databases) and can be read by imaging devices such as smart phone cameras.

**Figure 6:**
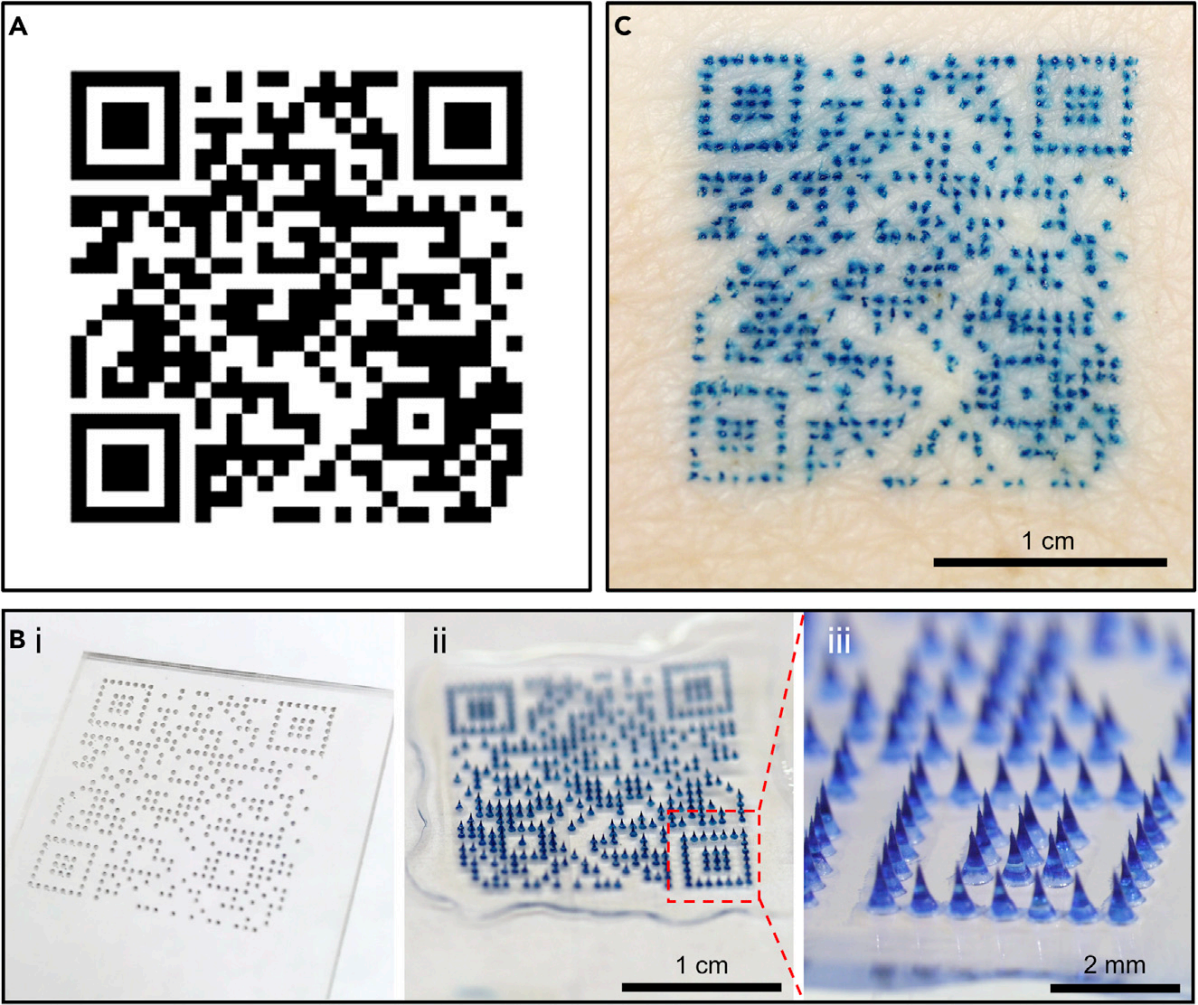
QR code tattoos by MN patches (A) Target 29 × 29 QR code encoding the website http://drugdelivery.chbe.gatech.edu/index.html. (B) The QR code pattern was (i) transferred to a PDMS mold, which was used to make a MN patch with a mirror image of the QR code: (ii) full patch, (iii) magnified view. (C) Representative photographic image of porcine skin with a QR code tattoo administered by MN patch *ex vivo*. The tattooed code was readable by QR code reader applications.

Our target QR code had 29 × 29 modules that encoded the URL address of the home page of our laboratory (Figure 6A). Like the other tattoo MN patches, we cast blue tattoo ink onto a PDMS mold with the QR code pattern to make an MN patch (Figure 6B) that, when applied to skin, transferred a tattooed pattern that matched the matrix of the target QR code (Figure 6C).

The tattooed dots were not square and were not immediately adjacent to each other, like standard QR code modules. Despite these differences, as well as visual and morphological features of the skin, the tattooed QR code was successfully read by various well-known applications, including “WeChat” (ver. 8.0.15, Tencent, Shenzhen, China) and “Tik Tok” (ver. 22.8.4, TikTok Pte., Singapore), as well as other widely installed code readers (>10,000,000 installs) from Google Play Store, such as “QR Code & Barcode Scanner” (ver. 2.5.2, QR Easy, Ho Chi Minh City, Vietnam), “QR & Barcode Scanner” (ver. 2.2.18, Gamma Play, Hong Kong, China), and “QR Scanner: Barcode Scanner & QR Code Scanner” (ver. 2.4.5.GP, Simple Design, British Virgin Islands); all readings tracked to our home page as expected.

Compared with simpler tattoo patterns, creating a QR code by MN patch has the advantage of storing much more information or providing a link to essentially unlimited information on the cloud. Disadvantages of QR code tattoos include their greater complexity requiring correct application of a larger patch to skin with high fidelity, although QR codes have built-in redundancy that enables correct reading even when part of the code image is obscured or lost. Interestingly, reading a QR code tattoo with UV illumination in the dark produces an inverted QR code with bright modules on a dark background, which may require (or enable) specialized code scanners.

### Applications of microneedle patch tattoos

Tattoos are in widespread use around the world for cosmetic, medical/veterinary, and other purposes. In current practice, administering a tattoo requires a person with specific technical, and in some cases artistic, skill. The process of administering a tattoo is time-consuming, painful, and risks causing infection (Klügl et al., 2010; Laux et al., 2016; Liszewski et al., 2015; Paola et al., 2016). As an alternative to current tattooing practice, MN patch tattoos could for some applications facilitate increased access to tattoos, reduce their cost, and increase their safety.

Cosmetic tattoos often cover large areas of skin and require customized artistry, but small tattoos with simple designs could be given quickly and painlessly by MN patch. Self-administration may also be possible, and use of temporary tattoo ink (which we did not explore in this study) may be especially suitable in these situations (Shah and Pierre, 2021). MN patches could also increase the safety of tattooing, because they can be manufactured sterile, are single-use, and generate no biohazardous waste (because the MNs dissolve in the skin) (Duarah et al., 2019; Ita, 2017; Prausnitz, 2017). MNs have been used in dozens of clinical trials for drug delivery and other applications with an excellent safety record (Bhatnagar et al., 2017) and have been sold for cosmetic purposes like anti-aging for decades (Iriarte et al., 2017). MN patches are strongly preferred over hypodermic injections (Arya et al., 2017; Gill et al., 2008; Haq et al., 2009), which are in turn much less painful than getting a tattoo that involves repeated needle injections. MN patch tattoos could reduce the cost of tattoos, because the patches are expected to cost less than USD $1 each, and there is no need for expert and time-consuming tattoo application using complicated tattooing devices typically available only at specific tattooing facilities, all of which may enable self-administration (Duarah et al., 2019; Ita, 2017; Prausnitz, 2017). These features of MN patch technology suggest that MN patch tattoos would be better accepted by the general population compared with conventional tattooing.

Medical tattoos can be very small and simple, such as those used to identify body sites for repeated treatments like radiotherapy (Landeg et al., 2016) or for veterinary tattoos identifying sterilization status of animals (Griffin et al., 2020). A MN patch tattoo may be ideally suited in such scenarios. More complex but relatively standardized designs like nipple-areola reconstruction therapy (Vandeweyer, 2003) or dermatographic color correction of vitiligo (Singh and Karki, 2010) could also be amenable to MN patch tattooing, applied over a larger area with multiple patches and with customized coloring based on the patient’s skin tone.

Many patients with chronic conditions wear medical alert bracelets, which in some cases could be replaced with tattoos (Kluger and Aldasouqi, 2013). In this study, we evaluated MN patch tattoos to provide information on blood type or diabetes status, but many other health care scenarios could be addressed, including medical conditions (asthma, epilepsy), medications (blood thinner, allergies), medical devices (pacemaker), do-not-resuscitate orders, and contact information.

MN patch tattoos could be useful in public health, especially in settings where health care resources and medical recordkeeping are limited. Information about an important medical intervention could be recorded by a small, discreet tattoo. We presented an example of a MN patch tattoo used to indicate the year of IPV vaccination, and this tattoo was combined into a two-component patch with a second MN array used to administer the vaccine. This approach is reminiscent of the small scars left in the skin by smallpox vaccination, which served as evidence of vaccination status to identify unvaccinated people during vaccination campaigns for smallpox eradication (Fenner et al., 1988).

MN patch tattoos could also provide dynamic information in response to environmental changes. Here, we showed tattoos that responded to light and heat, but there is a large body of literature on materials with properties that respond to a broad variety of physical and molecular inputs. Probably the best-known examples are glucose-responsive materials that change color or appearance to report glucose levels of interest to people with diabetes (Wu et al., 2011). However, there are many other materials that respond to change of pH, enzymes, binding reactions, redox state, ionic strength, mechanical stress, or other stimuli (Deirram et al., 2019; Kowalski et al., 2018; Linsley and Wu, 2017; Mu et al., 2018; Municoy et al., 2020; Ward and Georgiou, 2011; Wu et al., 2011) that could be formulated in particle form and administered to the skin as a MN patch tattoo. Such an environmentally responsive tattoo could be a convenient, discreet method for patients to continuously monitor their health status.

Larger MN patch tattoos can contain more information, either for direct readout of words, numbers, or symbols or for electronic readout, such as using a QR code that contains digitized information including a possible Internet site link. Although QR codes are standardized, other scanning codes could be used as well when combined with a suitable electronic reader.

In conclusion, this study developed MN patches to administer tattoos to the skin and demonstrated their feasibility for a variety of medical and other applications. The MN patches were designed to simplify the tattooing process by reducing the need for technical and artistic skill to administer tattoos and to increase acceptability of the process by tattoo recipients by reducing time and eliminating pain to improve tolerability. MN patch tattoos can also increase safety, as single-use, sterile, skin patches that generate no biohazardous sharps waste. The tattoos created in this way were used to record medical information, such as medical conditions and vaccination status, on the skin for use by emergency response and other health care personnel, using both light-visible and UV-visible inks. The MN patch tattoos were also capable of being responsive to environmental cues, such as light or temperature, which could be used to monitor physiological processes *in situ* in the body for medical, research, or other purposes. MN patch administration of complex tattoo patterns like QR codes further showed the breadth of possible applications. We conclude that MN patch tattoos can simplify tattoo administration, thereby increasing safety and access to tattoos for medical, cosmetic, research, and other uses.

### Limitations of the study

The potential use of MN patches for tattooing has limitations, considering both medical and cosmetic applications. Image resolution is constrained by MN density, which in this study was ∼100 MNs/cm^2^, but other patches have been developed with densities greater than 20,000 MNs/cm^2^ (Chen et al., 2010). Typical MN patch fabrication methods fill or coat all MNs with the same materials, which generates single-color tattoos, but novel approaches are under development to allow each MN to deliver different cargo (Allen et al., 2016; Li et al., 2018), which will enable multi-color tattoos. Tattoo size is limited by MN patch size, which currently is usually ∼1 cm^2^, limited largely by reliable insertion of the MNs into the skin, but there are examples of larger patches (Li et al., 2021; Ripolin et al., 2017). Even larger tattoos may require the use of multiple patches. Some of these limitations may be more critical to cosmetics, where aesthetic appeal of the tattoo is often most important, as opposed to medical tattoos, where functionality is the main goal. Another consideration is that skin texture and thickness at different body locations and on humans versus various animals might influence MN patch penetration and overall performance, thereby affecting the quality of tattoo images.

In some cases, MN patch tattoos could be administered by health care personnel or other professionals. However, MN patches currently used for cosmetics and drug delivery are self-administered by users with little or no training (Bhatnagar et al., 2017; Huang et al., 2022), which suggests that MN patch tattoos could likewise be self-administered or at least applied by people without specialized training. Because conventional tattoo inks are designed to be permanent, application of MN patch tattoos by people without training could lead to mistakes with lasting cosmetic consequences. Although tattoo removal is possible (Bäumler and Weiß, 2019), temporary tattoos (Shah and Pierre, 2021) may be a useful option for self-administration.

The ease of administration by MN patches facilitates new and more-widespread application of medical tattoos, which may raise privacy concerns when sensitive personal medical information is associated with a tattoo. On the one hand, storing private information in one’s own skin provides the ultimate personal control and security that no hacker can access over the internet. On the other hand, tattoos may be seen by unintended viewers, depending on body location. Tattoos can be made more discreet by covering with clothing or using inks that require UV, infrared, or other specialized optics. However, such barriers to access may limit the utility of tattoos used, for example, as medical alerts that emergency personnel need to easily find and read.

Future studies should further explore the range of MN patch tattoos in terms of image resolution, colors, size, and information content. MN patch tattoos should also be applied to humans to confirm their expected painlessness and acceptability, as well as their safety and longevity in the skin. The possibility of administering temporary or reversible tattoos by MN patch should be considered too. Issues related to privacy need to be evaluated, which will depend on the population, purpose, and other factors associated with the specific application.

## STAR★Methods

### Key resources table

**Table:** 

REAGENT or RESOURCE	SOURCE	IDENTIFIER
**Antibodies**		

Poliovirus Type 2 Monoclonal Antibody	ThermoFisher	Catalog #: HYB 294-06-02; RRID: AB_667506

**Chemicals, peptides, and recombinant proteins**		

SYLGARD™ 184 Silicone Elastomer Kit	Dow Corning	https://www.dow.com/en-us.html
Polyvinyl alcohol 4–88	Millipore Sigma	CAS: 9002-89-5
Polyacrylic acid (MW ∼50000)	Polysciences	CAS: 9003-01-4
Sucrose	Sigma-Aldrich	CAS: 57-50-1
Maltodextrin (dextrose equivalent 13.0 - 17.0)	Sigma-Aldrich	CAS: 9050-36-6
Xylitol	Sigma-Aldrich	CAS: 87-99-0
Dry powdered tattoo ink (Navy blue TN15)	National Tattoo Supply	Product Code: TN15-1-2
Dry powdered tattoo ink (Chinese red TN31)	National Tattoo Supply	Product Code: TN31-1-2
Dry powdered tattoo ink (Indigo black TN1)	National Tattoo Supply	Product Code: TN1-1-2
Blacklight UV invisible tattoo ink	Bloodline Tattoo Inks	https://bloodlineink.com/
Thermochromic pigment powder (blue to green)	UniGlow Products	www.uniglowproducts.com
Thermochromic pigment powder (violet to blue)	UniGlow Products	www.uniglowproducts.com
Thermochromic pigment powder (red to yellow)	UniGlow Products	www.uniglowproducts.com
Inactive Polio vaccine (IPV) type 2 (Middle East Forces (MEF) strain)	Bilthoven Biologicals (Bilthoven, Netherlands)	https://www.bbio.nl/

### Resource availability

#### Lead contact

Further information and requests for resources and reagents should be directed to and will be fulfilled by the lead contact, Mark R. Prausnitz (prausnitz@gatech.edu).

#### Materials availability

This study did not generate new or unique reagents.

### Experimental model and subject details

The rat study reported here was approved by the Institutional Animal Care and Use Committee (IACUC) of the Georgia Institute of Technology. Wistar rats (8–10 weeks old, female, Charles River Laboratories, Wilmington, MA) were used. Rats were housed at 22–25°C on a circadian cycle of 12-h light and 12-h dark with free access to food and water.

### Method details

#### Fabrication of MN patch molds

PDMS molds in the inverse shape of MN patches were made by laser cutting. PDMS sheets were prepared by mixing the two precursors of Sylgard 184 (Dow Corning, Midland, MI) at a ratio of 10:1. The mixture was degassed, poured into a flat-bottom container to a thickness of ∼2.5 mm, and cured at room temperature (20–25 °C) for 2 d and at 37°C for another day. A solid PDMS sheet was drilled by a CO_2_ laser cutter in vector mode in the VersaLaser engraver VLS 3.50 (Universal Laser Systems, Scottsdale, AZ) to generate an array of cone-like cavities to form the MNs in the PDMS mold. The MN dimensions and position were controlled by drawing in AutoCAD software (Autodesk, San Rafael, CA) so that each MN had a ∼550 μm base diameter, ∼1.1 mm length and tapered to a tip of ∼10 μm radius of curvature.

To clean the mold, a 20% (w/v) polyvinyl alcohol (PVA, 4–88, Millipore Sigma, Burlington, MA) solution in deionized water was applied on the drilled mold under vacuum and allowed to dry to form a PVA film at 40°C overnight. Peeling off the PVA film removed burnt debris on the PDMS mold which had been generated during laser drilling. The PDMS molds were stored at room temperature until use.

#### Fabrication of tattoo MN patches

Tattoo MN patches were fabricated using a two-step molding process in PDMS molds based on established methods (Edens et al., 2015a; Mistilis et al., 2015). Briefly, tattoo ink dry powder was dispersed at a concentration of 5% (w/v) in aqueous solution containing 10% (w/v) PAA (Polysciences, Warrington, PA) with the help of bath sonication. This dispersion was used as the first-cast solution to fill into the PDMS mold cavities under vacuum for 20 min at room temperature to form the ink-loaded MNs. Excess liquid was removed from the mold surface after this step.

The second casting solution, consisting of 18% (w/v) PVA and 18% (w/v) sucrose (Sigma, St. Louis, MO), was then applied on the molds and dried under vacuum at room temperature for 3 h to form the backing layer of MN patches. The filled molds were further dried at 40°C overnight before demolding the MN patches using adhesive tape (Scotch, 3M, St. Paul, MN). All MN patches were stored with desiccant at room temperature until further use.

Different types of tattoo inks or pigments were loaded into the MN patches to enable different tattoo appearance and function. The inks included (1) visible-colored dry tattoo powers (Navy blue TN15, Chinese red TN31 and Indigo black TN1, National Tattoo Supply, Allentown, PA), (2) UV-visible tattoo ink (Blacklight Invisible, Bloodline, Carson City, NV) and (3) thermochromic ink that changes color when heated above 31°C (UniGlow, Tampa, FL). These inks were loaded in MN patches to get solid-color MN patches, UV-MN patches, and thermo-MN patches, respectively. To fabricate MN patches, dry ink powders were added directly and suspended in casting solutions. For the liquid UV ink, the ink was centrifuged at ∼5000 × g for 3 min to separate the ink particles. The ink particles were then washed with an equal volume of DI water and dried at 37°C for 2 days to yield dry ink powder.

#### Application of MN patches to tattoo skin

The feasibility of tattooing skin by MN patches was examined by applying tattoo MN patches to excised porcine skin *ex vivo* and to shaved rats *in vivo*. Briefly, MN patches were pressed by thumb against the skin for ∼10 s and left in place for 15 min prior to peeling off. After application, the skin was gently cleaned by tissue paper (Kimwipe, Kimberly-Clark Professional, Roswell, GA) to wipe off residual ink and MN patch dissolution products from the skin surface. Photographs of the tattooed skin were taken by digital camera (Canon EOS 60D, Tokyo, Japan) from 20 cm or 1 m away.

When applying tattoo MN patches *in vivo*, Wistar rats (8–10 weeks old, female, Charles River Laboratories, Wilmington, MA) were anesthetized by isoflurane and shaved to exposure at least an 8 cm × 5 cm hairless area for MN patch application and photographing afterwards. The tattoos were imaged by camera photography immediately after applying the MN patches and every month after that. All procedures in this study involving animals were approved by the IACUC at the Georgia Institute of Technology.

To visualize and photograph UV tattoos made by MN patches, a portable UV flashlight (TT-FL002, TaoTronics, Shenzhen, China) was used to illuminate the tattooed skin area from ∼0.5 m away.

#### Fabrication of tattoo MN patches for vaccination

Two-component MN patches were designed to deliver IPV vaccine and to make tattoo markers at the same time. Monovalent bulk IPV type 2 (Middle East Forces (MEF) strain) was kindly provided by Bilthoven Biologicals (Bilthoven, Netherlands). The antigen concentration in the bulk vaccine was measured to be 834 D-antigen units per milliliter (DU/mL). The stock IPV solution was concentrated with Amicon ultracentrifuge spin filters with 100 kDa molecular weight cutoff (Millipore Sigma). The final antigen concentration was determined to be 3.6 kDU/mL.

All DU values were measured with sandwich enzyme-linked immunosorbent assay (ELISA) using an established method (Edens et al., 2015b). Briefly, 50 μL of diluted antibody (poliovirus type 2 monoclonal antibody, ThermoFisher, Waltham, MA) were dispensed into Nunc MaxiSorp high protein-binding capacity 96-well plates (ThermoFisher) and incubated at 4°C for 1 day to coat plates. The coated plates were washed four times with wash buffer (0.01 M PBS, pH 7.2 with 0.05% Tween 20) and blocked with blocking buffer (0.01 M PBS, pH 7.2 with 0.25% Tween-20 and 0.5% gelatin) at 37°C for 1 h. The blocked plates were washed four times and then received 50 μL of antigen for incubation at 37°C for 1 h. After washing plates four times, 50 μL of diluted horseradish peroxidase (HRP) labelled antibody was added and incubated at 37°C for 1 h. After washing plates, 50 μL of SureBlue Reserve TMB Microwell Peroxidase Substrate (1-Component) (KPL, Gaithersburg, MD) was added and incubated at 37°C for 15 min, and the reaction was stopped by adding 50 μL of TMB BlueSTOP Solution (KPL). Absorbance at 620 nm was evaluated on a spectrophotometer to analyze plates.

The IPV vaccine and tattoo ink were loaded separately into different sections of the MN patch to obtain two-component MN patches. The tattoo ink was loaded into one side of the MN patch as described above. The IPV was similarly loaded into the other side of the MN patch but with a different first-cast solution containing 3.6 kDU/mL IPV, 7.5% (w/v) maltodextrin (Sigma) and 2.5% (w/v) xylitol (Sigma), and a different second-cast solution containing 36% (w/v) maltodextrin, 12% (w/v) xylitol and 1% (w/v) PVA. The two second-cast solutions contacted each other on the mold surface and were dried into a single patch.

#### Application of MN patches to vaccinate and tattoo skin

Wistar rats (9 weeks old, female, Charles River Laboratories) were anesthetized via isoflurane inhalation during MN patch applications. Five rats were immunized against IPV type 2 with the two-component MN patch that administered 8 DU IPV vaccine to the skin. MN patches were pressed against shaved rat skin for ∼10 s and removed after 15 min. For the control group, 6 rats were immunized against IPV type 2 by intramuscular (IM) injection in the thigh muscle of the hind limb. IM injection solutions were prepared by reconstituting and diluting the vaccine portion of a MN patch to deliver the same dose in a 100 μL IM injection.

Blood samples from the animals were collected over a six-week period to determine polio-specific neutralizing antibody titers. All blood samples were collected via tail-vein bleeding in collection tubes containing clot activators (BD Diagnostics, Franklin Lakes, NJ). Serum from the blood samples was separated via centrifugation at 6000 × g for 1.5 min. Serum samples were stored in Eppendorf tubes at −20°C until antibody titer analysis. All procedures in this study involving animals were approved by the IACUC at the Georgia Institute of Technology.

Polio-specific neutralizing antibody titers in serum samples were determined by the Division of Viral Diseases, National Center for Immunization and Respiratory Diseases at Centers for Disease Control and Prevention (Atlanta, GA) using an established method (WHO, 1996). Briefly, 80–100 cell culture infectious dose 50% (CCID_50_) of poliovirus type 2 and serially diluted serum samples were mixed and incubated at 35°C for 3 h, followed by the addition of Hep-2 cells. After 5 days of incubation at 35°C and 5% CO_2_, the plates were stained with crystal violet, and the cell viability was determined by optical density measurements in a spectrophotometer. Then, the neutralization titer was calculated via the Spearman-Karber method (Kärber, 1931). The limit of detection for the assay was a 2.5 log_2_ titer, and the precision of detection was ±0.5 log_2_ titer.

### Quantification and statistical analysis

We report the average values of neutralizing antibody titers after IPV vaccination, and standard deviations of the results based on the measurements from 5–6 animals. An *F*-test was performed to examine the standard deviations between data sets prior to using a two-tailed Student’s *t* test to compare between groups. The difference was considered significant if p < 0.05.

## Data Availability

• Data: All data reported in the paper are available from the lead contact upon request. • Code: This paper does not report original code. • Any additional information related to this study is available from the lead contact upon request.
