# Supplementary material for: Microneedle patch tattoos
Source: iScience. 2022 Sep 14;25(10):105014. doi: 10.1016/j.isci.2022.105014 (PMC9617462; doi:10.1016/j.isci.2022.105014)
Supplement: Document S1. Figures S1 and S2 [file mmc1.pdf]

iScience, Volume *25*

Supplemental information

Microneedle patch tattoos

Song Li, Youngeun Kim, Jeong Woo Lee, and Mark R. Prausnitz

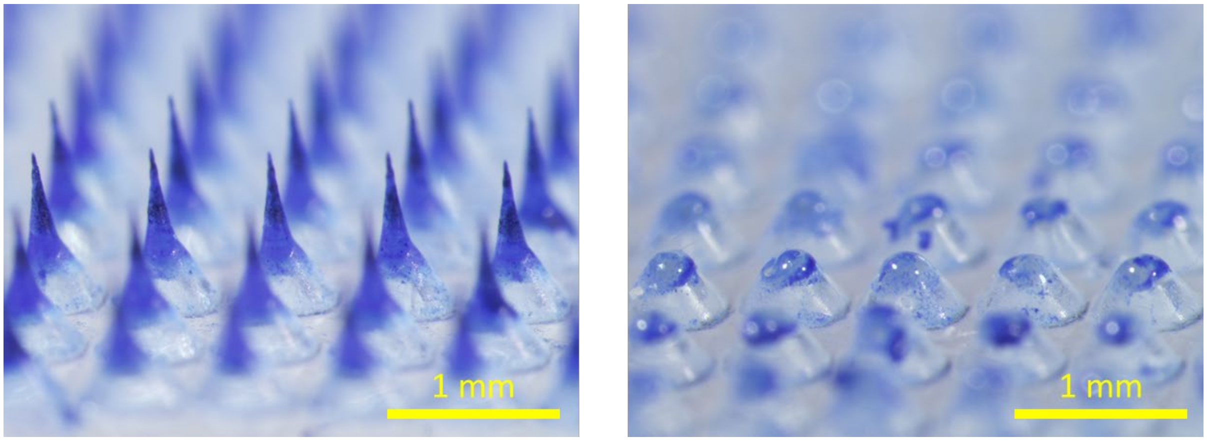

Figure S1. Representative microscopy images of MN patches before (left) and after (right) application to pig skin ex vivo, related to Figure 1A. Left photo: MN patch showing blue tattoo ink loaded in MNs. Right photo: MN patch shown after application and dissolution in the skin, which deposited tattoo ink in the skin and left behind a used patch backing with no biohazardous sharps.

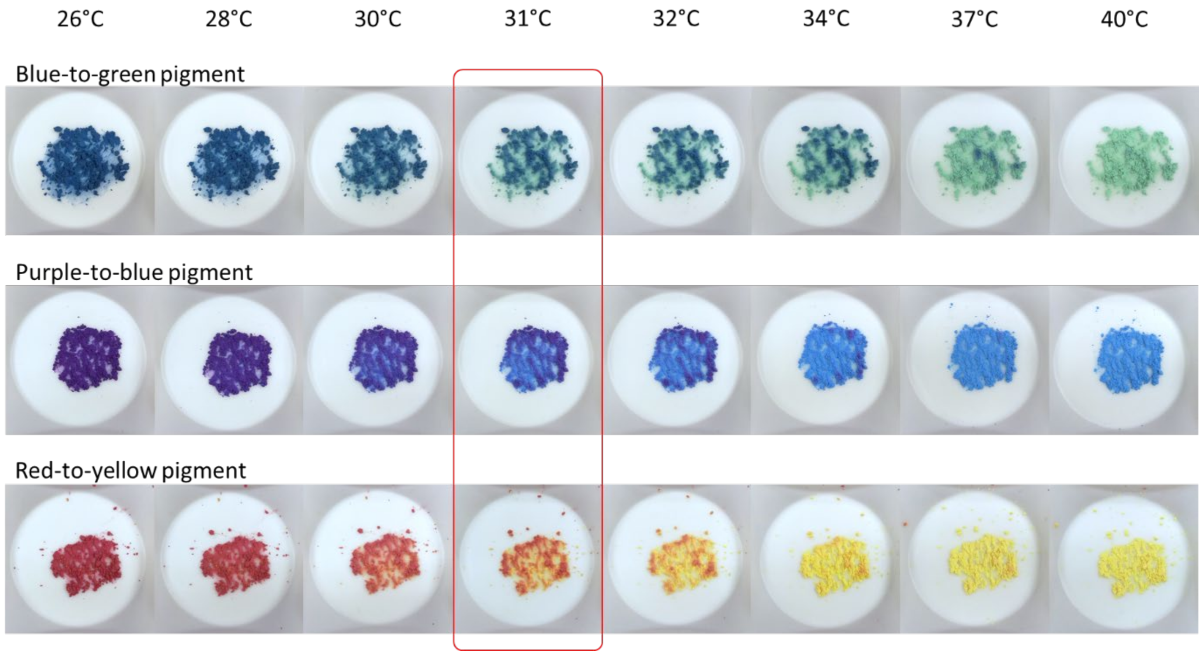

Figure S2. Examples of thermochromic pigments changing their colors upon heating, related to Figure 5. The color change of thermochromic pigments occurred primarily above 31 °C and was reversible when cooled. Top row: color of pigments changed from blue to green upon heating. Middle row: color of pigments changed from purple to blue upon heating. Bottom row: color of pigments changed from red to yellow upon heating.
